# A Ketogenic Diet in Combination with Gemcitabine Increases Survival in Pancreatic Cancer KPC Mice

**DOI:** 10.1158/2767-9764.CRC-22-0256

**Published:** 2022-09-08

**Authors:** Natalia E. Cortez, Cecilia Rodriguez Lanzi, Brian V. Hong, Jihao Xu, Fangyi Wang, Shuai Chen, Jon J. Ramsey, Matthew G. Pontifex, Michael Müller, David Vauzour, Payam Vahmani, Chang-il Hwang, Karen Matsukuma, Gerardo G. Mackenzie

**Affiliations:** 1 Department of Nutrition, University of California, Davis. Davis, California.; 2 Department of Microbiology and Molecular Genetics, University of California Davis, Davis, California.; 3 Department of Animal Science, University of California, Davis. Davis, California.; 4 Division of Biostatistics, Department of public Health Sciences, University of California, Davis. Davis, California.; 5 Department of Molecular Biosciences, University of California Davis, Davis, California.; 6 Norwich Medical School, Biomedical Research Centre, University of East Anglia, Norwich, United Kingdom.; 7 University of California, Davis Comprehensive Cancer Center, Sacramento, California.; 8 Department of Pathology and Laboratory Medicine, Sacramento, California.

## Abstract

Pancreatic ductal adenocarcinoma (PDAC) continues to be a major health problem. A ketogenic diet (KD), characterized by a very low carbohydrate and high fat composition, has gained attention for its antitumor potential. We evaluated the effect and mechanisms of feeding a strict KD alone or in combination with gemcitabine in the autochthonous LSL-Kras^G12D/+^; LSL-Trp53 ^R172H/+^; Pdx1-Cre (KPC) mouse model. For this purpose, both male and female pancreatic tumor-bearing KPC mice were allocated to a control diet (CD; %kcal: 65% carb, 15% protein, 20% fat), a KD (%kcal: 1% carb, 15% protein, 84% fat), a CD + gemcitabine (CG), or a KD + gemcitabine (KG) group. Mice fed a KD alone or in combination with gemcitabine showed significantly increased blood β-hydroxybutyrate levels compared with mice fed a CD or CG. KPC mice fed a KG had a significant increase in overall median survival compared with KPC mice fed a CD (increased overall median survival by 42%). Interestingly, when the data were disaggregated by sex, the effect of a KG was significant in female KPC mice (60% increase in median overall survival), but not in male KPC mice (28% increase in median overall survival). Mechanistically, the enhanced survival response to a KD combined with gemcitabine was multifactorial, including inhibition of ERK and AKT pathways, regulation of fatty acid metabolism and the modulation of the gut microbiota. In summary, a KD in combination with gemcitabine appears beneficial as a treatment strategy in PDAC in KPC mice, deserving further clinical evaluation.

**Significance:**

This article is the first preclinical study to comprehensively evaluate the effect of a KD alongside chemotherapy using a standard autochthonous genetically modified mouse model (in both male and female KPC mice).

## Introduction

Despite extensive efforts to develop new treatment strategies, pancreatic ductal adenocarcinoma (PDAC) continues to be a major health problem, with a 5-year survival of approximately 11% (1). While surgery is a viable option in a limited number of patients, the majority of patients with PDAC (>80%) are diagnosed with advanced, unresectable, or metastatic disease (2). For these patients, the standard treatments include combination of gemcitabine plus nanoparticle albumin bound (nab)-paclitaxel (Abraxane), or the combined therapy of leucovorin-modulated 5-fluorouracil, irinotecan, and oxaliplatin (FOLFIRINOX; refs. 2–5). Unfortunately, these chemotherapeutic strategies still provide limited clinical benefit. Hence, there is an urgent need to develop therapies that can improve outcomes in patients with PDAC, and the exploration of dietary interventions is a critical component.

During the last years, there has been considerable interest in the antitumor evaluation of ketogenic diets (KD; 6). KDs are characterized by a high fat, moderate protein, and very low carbohydrate content. These diets mimic changes in metabolism that are similar to fasting by elevating circulating levels of ketone bodies (i.e., acetoacetate, β-hydroxybutyrate, and acetone), which serve as an alternative energy source (7) and as signaling molecules (8). Numerous studies have indicated that a KD inhibits tumor growth and increases survival (9–11), including in PDAC (12–14). Multiple cellular mechanisms might explain the beneficial effects of a KD in tumor growth. These include anti-inflammatory, antiangiogenesis, cell metabolism, and epigenetic effects, as well as modulation of the microbiome (15). Unfortunately, many of these studies were performed using xenograft models of pancreatic cancer, which do not closely recapitulate human PDAC, so their clinical significance is limited (16). Recently, Yang and colleagues, reported that a KD was effective as an chemotherapy adjuvant reducing tumor growth in syngeneic subcutaneous pancreatic tumors and prolonged survival in the clinically relevant *LSL-Kras^G12D/+^*, *LSL-Trp53^R172H/+^*, *Pdx1-Cre* (KPC) genetically engineered mouse model of pancreatic cancer (17). However, this study was performed using a small cohort of only male mice.

In this study, we evaluated the impact of feeding a strict KD alone or in combination with gemcitabine in the autochthonous and clinically relevant KPC mouse model of pancreatic cancer (18, 19). Furthermore, we examined whether there might be sex-related differences in the response to a KD in PDAC. We observed that a KD in combination with gemcitabine extends survival in KPC mice and that female mice appear to be slightly more responsive to the KD. The mechanisms by which a KD plus gemcitabine increases survival response appear to be multifactorial, including inhibition of ERK and AKT pathways, regulation of fatty acid metabolism, and the modulation of the gut microbiota.

## Materials and Methods

### Animal Studies

All animal used procedures were approved by the University of California, Davis Animal Care and Use Committee.

### Genetically Engineered Transgenic Mice

The genetically engineered LSL-KrasG12D/+; LSL-Trp53R172H/+; Pdx-1-Cre (KPC) mice were bred at the UC Davis Animal Facility in Meyer Hall. KPC mice were generated from three mouse parental strains (LSL-KrasG12D/+; LSL-Trp53R172H/+; and Pdx-1-Cre), obtained from NCI mouse repository, following established procedures described by Hingorani and colleagues (18). After weaning, mice were individually housed in polycarbonate cages in a room with controlled temperature (22°C–24°C) and humidity (40%–60%), maintained on a 12-hour light-dark cycle, and fed chow diet ad libitum LabDiet 5001 (LabDiet) until enrolled in the studies.

### Survival Study

**FIGURE 1:**
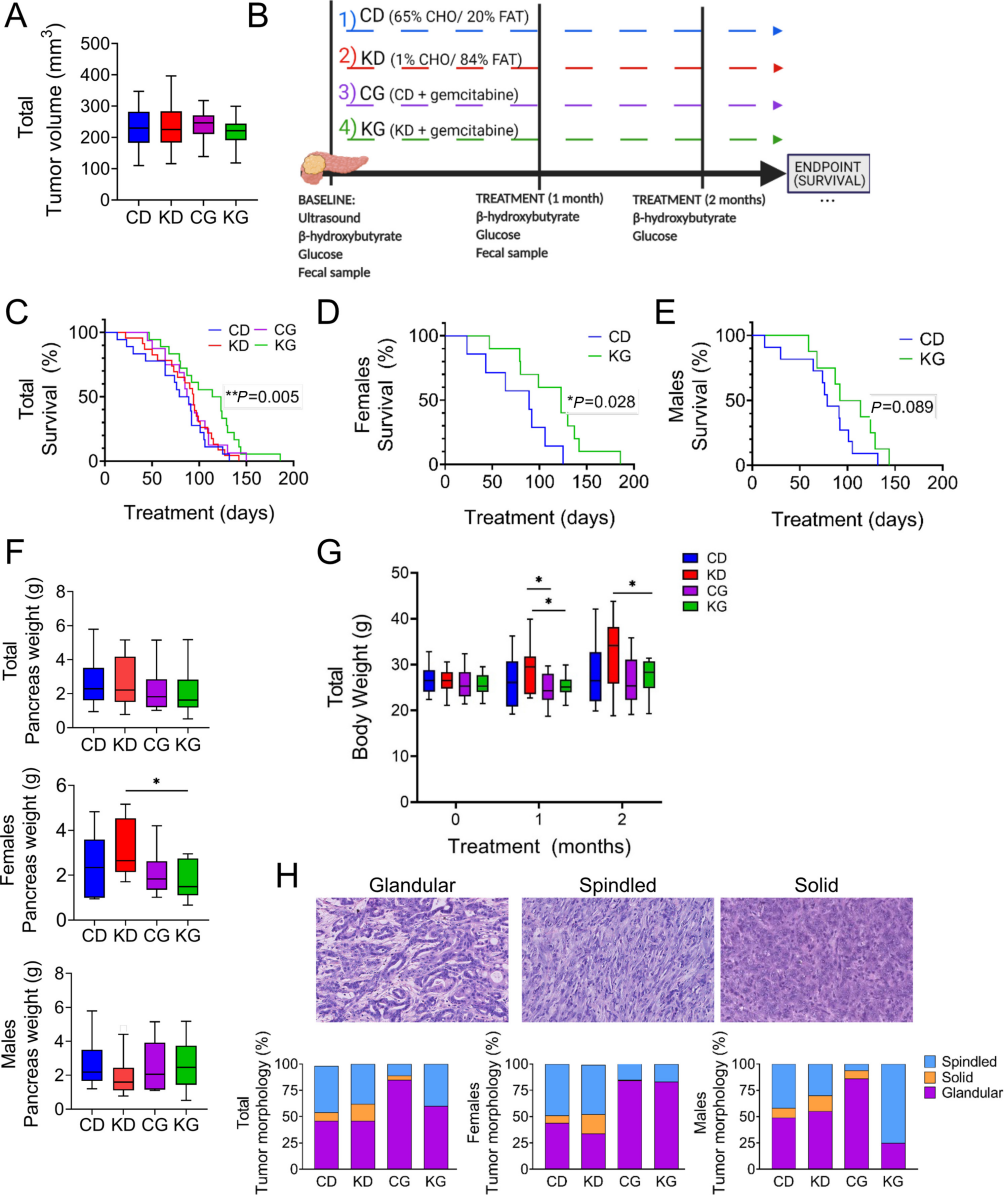
A KG extends median overall survival in KPC mice. **A,** Mean tumor volume of KPC mice at enrollment. **B,** Schematic outline of the survival study design. **C,** Kaplan–Meier survival curves of male and female KPC mice fed a CD, KD, CG or KG. **D** and **E,** The effect of KG extending median overall survival was more pronounced in female KPC mice than in male KPC mice. Of note: KD and CG groups had no significant effects, compared with CD group, and are not displayed for clarity. **F,** Pancreatic tumor weight at endpoint for total cohort (left), females only (center), and males only (right) are shown. *, *P < 0.05*. **G,** Body weight progression. **H,** Histopathologic analysis of pancreatic tumor morphology isolated from female and male KPC mice treated with CD, KD, CG, or KD. Tumor morphology was predominantly glandular. However, spindled and solid patterns were also observed, as described previously (18). Representative images of morphologic patterns: Glandular (left), Spindled (center), Solid (right). Hematoxylin and eosin–stained sections. All images digitally scanned at 20 × original magnification.

Enrollment of KPC mice was based on tumor size, measured using a high-resolution ultrasound imaging of the pancreas with the Vevo 2100 System with a 35 MHz RMV scan-head (Visual Sonics, Inc.), when KPC mice were around 3–4 months old (Supplementary Fig. S1A). Imaging was obtained and tumor volumes measured following previously published guides (20, 21). Once tumor size was assessed (Fig. 1A), male and female KPC mice were assigned randomly to one of four groups: a control diet (CD), KD, a control diet plus gemcitabine (CG) or a ketogenic diet plus gemcitabine (KG).

### Dietary Interventions

Following tumor size determination, male and female KPC mice (7–12 mice per sex per group; 16–23 mice/group) were allocated to either a CD (%kcal: 65% carb, 15% protein, 20% fat), a KD (%kcal: 1% carb, 15% protein, 84% fat), a CD + gemcitabine, or a KD + gemcitabine group. Mice were fed *ad libitum,* and food was changed and food intake was recorded three times per week. The composition of diets was adapted from the study by Roberts and colleagues (22), and is shown in Supplementary Table S1. The Envigo mineral mix TD94046 was used for the CDs and the TD79055 was used for the KDs due to their lower carbohydrate contents. For both diets, TD40060 (vitamin mix) was used.

### Chemotherapy Treatment

Gemcitabine (>99% 2′-Deoxy-2′,2′-difluorocytidine; dFdC; Gemzar; LY-188011) from Thermo Fisher Scientific was administered to the CG and KG groups at 100 mg/kg by intraperitoneal injections twice per week for 3.5 weeks (seven total injections).

Throughout the survival study, mice were observed daily for signs of significant weight loss, hemorrhagic ascites, and for other signs of clinical failure including loss of thermoregulation, inactivity, and presence of malignant ascites. Endpoint criteria included the development of abdominal ascites, weight loss exceeding 20% of the initial weight, or extreme weakness or inactivity. When an animal reached the endpoint criteria, it was euthanized by carbon dioxide asphyxiation, blood was collected and tissues, including pancreatic tumors were dissected, weighted, and then stored in liquid nitrogen, RNA later, and 10% buffered formalin.

### Blood Glucose and Ketones

Nonfasting glucose levels were measured using a glucometer (Easy Plus II, Home Aid Diagnostics Inc), and β-hydroxybutyrate levels were measured using the Precision Xtra glucose and ketone monitoring system (Abbott) according to the manufacturer's instructions.

### Mechanistic Study

A cohort of mice was allocated to either the CG or the KG groups after tumor detection and euthanized at 2 months after interventions. We chose CD + gemcitabine as our control group to specifically depict the contribution of a KD to the effect. At the end of the 2 months, pancreas and pancreatic tumors were dissected, weighed, sectioned, and then stored in liquid nitrogen, RNAlater, and 10% buffered formalin.

### Metabolic Measurements

Blood samples were collected via cardiac puncture and serum was isolated after centrifugation at 3,000 × *g* for 10 minutes at room temperature. Insulin was assayed using the V-PLEX mouse metabolic kit and mouse leptin kit. Inflammation-related biomarkers were assayed using the V-PLEX Proinflammatory panel I kit (Meso Scale Discovery).

### Histology

After necropsy, pancreas specimens were fixed in 10% buffered formalin overnight at 4°C. Tissues were processed and embedded by routine methods. Tissue sections (5 μmol/L) were stained with hematoxylin and eosin or Masson's Trichrome (Chromaview, Thermo Fisher Scientific). Tumors were classified by morphologic pattern (glandular, spindled, solid), and each morphologic pattern was scored as a percentage of total tumor surface area. Presence and extent of tumor necrosis, and presence and type of background pancreatic fibrosis (e.g., interlobular, intralobular) were also scored. All histologic sections were evaluated in a blinded fashion.

### IHC Staining

Pancreas were fixed in 10% buffered formalin overnight at 4°C, processed and embedded using routine methods. Paraffin sections were deparaffinized, rehydrated, and heated for 12 minutes at 95°C in 10 mmol/L (pH 6) citrate buffer (M-15704, Thermo Fisher Scientific). Afterward, sections were incubated with 3% hydrogen peroxide (59105926, Millipore corporation) for 10 minutes and blocked-in animal-free Blocker (SP-5030, Vector laboratories) for 1 hour at room temperature and then incubated overnight at 4°C with primary antibody against p-ERK1/2 (1:200 dilution, Cell Signaling Technology, catalog no. 4376, RRID:AB_331772). The following day, paraffin sections were incubated with biotin-conjugated secondary antibody for 1 hour at room temperature (856743, Life Technologies), horseradish peroxidase (HRP) streptavidin for 1 hour at room temperature (856743, Life Technologies), and developed by DAB (SK-4100, Vector Laboratories), followed by hematoxylin (MHS16, Sigma) staining. Sections were then dehydrated, mounted in Cytoseal 60 mounting medium (8310-16, Thermo Fisher Scientific), and analyzed using an Olympus BX51 microscope. *Scoring:* Five or more fields per sample (at magnification × 200) were scored and the percent of positive cells was calculated as described previously (23).

### Western Blot Analysis

Pancreas tissue homogenates were prepared and Western blots were performed as described previously (23). Aliquots of total homogenates containing 25–40 μg protein were separated by reducing 8%–12.5% (w/v) PAGE and electroblotted onto nitrocellulose membranes. Membranes were blocked in 5% (w/v) nonfat milk for 1 hour and subsequently incubated with the following antibodies from Cell Signaling Technology: p-ERK (catalog no. 4376, RRID:AB_331772), ERK (catalog no. 9102, RRID:AB_330744), p-Akt (Ser473; catalog no. 4060, RRID:AB_2315049), AKT (catalog no. 9272, RRID:AB_329827), p-AMPKα (Thr172: catalog no. 2535, RRID:AB_331250), AMPKα (catalog no. 2532,RRID:AB_330331), p-4E-BP1 (Thr37/46; catalog no. 2855, RRID:AB_560835), 4E-BP1 (catalog no. 9452, RRID:AB_331692), HKII (catalog no. 2867, RRID:AB_2232946), PDH (catalog no. 3205, RRID:AB_2162926), LDH (catalog no. 2012, RRID:AB_2137173), p-IGFR-R (catalog no. 3024, RRID:AB_331253), IGFR-R (catalog no. 9750, RRID:AB_10950969), and PKM2 (catalog no. 4053, RRID:AB_1904096), using a 1:1,000 dilution, overnight at 4°C. After incubation for 1 hour at room temperature in the presence of secondary antibodies (either HRP or biotinylated antibodies, followed by 1 hour incubation with streptavidin when biotinylated antibody was used in a 1:5,000 dilution), the conjugates were visualized and quantified by chemiluminescence detection in a Chemidoc Imaging-System, Bio-Rad Laboratories (RRID:SCR_008426), Inc. β-Actin (catalog no. A1978) from Millipore-Sigma, was used as a loading control. The densitometry analysis was performed using the Image J Program (RRID:SCR_003070).

### RNA Preparation and RNA Sequencing Analysis

Mice for the RNA sequencing (RNA-Seq) data were treated for 2 months with diet and chemotherapy. Tissues were stabilized in RNAlater (Thermo Fisher Scientific). Total RNA was extracted following the manufacturer's instructions using a RNeasy mini kit (74104, QIAGEN) from frozen pancreas and/or tumors. RNA quality was confirmed using Nano drop one (Thermo Fisher Scientific). Library preparation and RNA-Seq were performed by Novogene Co., LTD. In brief, mRNA was enriched using oligo(dT) beads, and rRNA was removed using the Ribo-Zero kit. The mRNA was fragmented, and cDNA was synthesized by using mRNA template and random hexamers primer, after which a second-strand synthesis buffer (Illumina), dNTPs, RNase H, and DNA polymerase I were added for the second-strand synthesis, followed by adaptor ligation and size selection. The library was sequenced by the Illumina Novaseq platform. Raw data were aligned to mm10 genome using HISAT2, read counts and normalized read count were generated using the feature counts, and the differentially expressed genes were identified using DESeq2 (RRID:SCR_000154).

### Tissue Fatty Acid Analysis

Fatty acid content in KPC pancreatic tumors of CG- and KG-treated mice was measured using gas chromatography (GC). Briefly, pancreatic samples were freeze dried and direct methylated with sodium methoxide as described previously (24). *Cis*-10–17:1 methyl ester (Nu-Check Prep Inc.) was added as an internal standard prior to methylating reagent. Fatty acid methyl esters (FAME) were analyzed by GC using a CP-Sil88 column (100 m, 25 μm ID, 0.2 μm film thickness) in a TRACE 1310 gas chromatograph (Thermo Fisher Scientific) equipped with a flame-ionization detector (GC-FID, Thermo Fisher Scientific). Each sample was analyzed twice by GC using a 175°C plateau temperature program (24). The FAME were quantified using chromatographic peak area and internal standard-based calculations.

### Microbiota Analysis

Fecal samples were collected from KPC mice at baseline and 1 month after dietary intervention ± gemcitabine treatment (from the KPC survival study groups). All fecal samples were collected directly from the animals on Eppendorf tubes and immediately frozen in liquid nitrogen. Genomic DNA was extracted from all samples using a commercially available kit (Qiagen QIAamp PowerFecal Pro DNA Kit, catalog no. 51804) and following manufacturer's instructions. DNA concentrations of each sample were evaluated using Qubit dsDNA High Sensitivity Assay Kit (catalog no. Q32851) with Qubit 4.0 Fluorometer, following manufacturer's instructions. Quality assessment was performed by agarose gel electrophoresis to detect DNA integrity, purity, fragment size, and concentration. The 16S rRNA amplicon sequencing of the V3-V4 hypervariable region was performed with an Illumina NovaSeq 6000 PE250. Sequences analysis were performed by Uparse software (Uparse v7.0.1001; 25), using all the effective tags. Sequences with ≥97% similarity were assigned to the same operational taxonomic units (OTU). Representative sequence for each OTU was screened for further annotation. For each representative sequence, Mothur software was performed against the SSUrRNA database of SILVA Database (26). OTUs abundance information was normalized using a standard of sequence number corresponding to the sample with the least sequences.

### Data Availability

The accession number for the RNA-Seq data reported in this study is NCBI Gene Expression Omnibus: GSE208398. The accession number for the microbiome data is under Bioproject number: PRJNA858994.

### Statistical Analysis

The data, obtained from at least three independent experiments, were expressed as the mean ± SEM. Statistical evaluation was performed by *t*-test or one-factor ANOVA followed by the Tukey test adjusted for multiple comparisons. Analyses were performed by GraphPad (Prism version 9.2, RRID:SCR_002798) and R version 4.0.4. Two-sided *P* < 0.05 was regarded as statistically significant.

Kaplan–Meier methods and the log-rank tests were used to compare unadjusted survival outcome (time from the start of treatment to death) between treatments in overall and key subgroups. There is no censoring in the survival outcome. To adjust for possibly unbalanced age and sex between treatment groups and explore potential interactions, linear regression models for survival days since treatment to death were performed, which include diet (CD, KD), gemcitabine (no, yes), sex, age at the start of treatment (centered at 90 days), interaction between diet and gemcitabine, two- and three-way interactions between sex and treatments (diet and gemcitabine). All interactions were removed from the final model due to nonsignificance. Model diagnosis was performed to ensure that the assumptions of linear regressions hold.

For the microbiota analysis, alpha- and beta-diversity were assessed by using standard metrics (e.g., Simpson and Shannon H diversity index) and Bray–Curtis principal coordinates of analysis (PCoA), respectively. Statistical significance was determined by Kruskal–Wallis or permutational multivariate ANOVA (PERMANOVA). Comparisons at the Phylum and Genus level were made using classical univariate analysis using Kruskal–Wallis combined with a FDR approach used to correct for multiple testing. Finally, LEfSe (linear discriminant analysis effect size) was also employed to determine the features most likely to explain differences between classes.

## Results

### A KD Plus Chemotherapy Extends Survival in KPC Mice

We first conducted a survival study in the clinically relevant KPC mouse model to evaluate the effect of feeding a strict KD alone or in combination with gemcitabine as a treatment protocol in male and female KPC mice bearing pancreatic tumors. For this purpose, we enrolled KPC mice with similar tumor sizes, measured by high-resolution ultrasound imaging of the mouse pancreas (Fig. 1A; Supplementary Fig. S1). Male and female KPC mice were divided into CD, KD, CD + gemcitabine (CG), or KD + gemcitabine (KG) groups (16–23 mice/group; Fig. 1B). While a KD alone had no significant effect on KPC survival, the combination of a KD with gemcitabine synergistically prolonged survival. The overall median survival times among the four groups were 80, 94, 88, and 119 days for CD, KD, CG, and KG groups, respectively. While KD alone or CG treatments were unable to extend KPC mouse survival, KPC mice fed a KG had a significant increase in overall median survival compared with KPC mice fed a CD (increased overall median survival by 42%; Fig. 1C). A 26% increase survival was observed when comparing KG group with CG. Interestingly, when we separated by sex, the effect of a KG was significant in female KPC mice (60% increase in median overall survival (*P* = 0.028), but not in male KPC mice [28% increase in median overall survival compared with CD mice (*P* = 0.089),] (Fig. 1D and E; Supplementary Fig. S1B). The median survival times for CD and KG groups were 77 and 123 days in females; and 80 and 103 in males, respectively. Interestingly, the weights of the pancreas/tumors were comparable among the groups, with only a significant decrease of the tumor weight of KG-treated female mice was observed compared with KD alone (Fig. 1F). Of note, treatment with KD, CG, or KG was well tolerated with no body weight loss throughout the treatment, as compared with the baseline body weight levels (Fig. 1G).

Histopathologic evaluation of the tumors showed the classic glandular morphology of PDAC, as originally described by Hingorani and colleagues (18). Spindled and solid patterns were also observed as secondary or primary patterns [also described by Hingorani and colleagues (18); Fig. 1H]. Interestingly, an increased proportion of CG and KG tumors demonstrated classic glandular morphology compared with CD and KD tumors (Fig. 1H), whereas the latter showed an increased proportion of spindled and solid patterns, generally considered indicative of more aggressive behavior (27, 28). In addition, the increase in glandular morphology in the KG-treated group was found to be the result of a marked predominance of glandular morphology in the tumors of the female mice. Some tumors showed some degree of necrosis. Overall, we noted a slight increase in tumor necrosis in females fed the KD compared with CD, with and without gemcitabine (Supplementary Fig. S2). The residual background pancreas (when present) showed a combination of intralobular and interlobular fibrosis. No significant difference in the pattern of fibrosis (intralobular vs. interlobular) was noted among the four groups (Supplementary Fig. S3).

To adjust for possibly unbalanced age and sex between intervention groups and further explore whether a KD ± gemcitabine's survival effect is sex-dependent, we conducted linear regression models for survival days since treatment to death, which was adjusted by sex and age at the start of treatment. As shown in Table 1, gemcitabine significantly extended mean survival by 25.8 days (*P* = 0.002), KD extended mean survival by 13.8 days with a trend toward significance (*P* = 0.052). Compared with CD, KG significantly extended mean survival by 39.6 days (*P* < 0.001). Although Kaplan–Meier curves in sex subgroups suggested that the effect of KG is likely more effective in females than in males, the interactions between sex and treatments in linear regressions were not significant and hence removed from the final model. Thus, the treatment effect was comparable across both sexes, benefiting both females and males.

**TABLE 1:** Parameter estimates from the linear regression model for survival days since treatment to death

Model term	Estimate (SE)	*P*
Intercept	70.35 (8.83)	<0.001
Effect of Keto vs. Control	13.83 (7.01)	0.052
Effect of gemcitabine vs. No gemcitabine	25.76 (7.95)	0.002
Effect of age at the start of treatment (in days, centered at 90)	−0.25 (0.30)	0.406
Effect of male vs. female	−13.19 (7.07)	0.066

Note: Linear regression for survival days since treatment to death was adjusted by sex and age at the start of treatment. There is no censoring in data. Age at the start of treatment is centered at 90 days; thus, the intercept can be interpreted as the predicted survival days since treatment for a female animal taking conventional diet and no gemcitabine, with treatment starting at 90-day old. The estimate for age at the start of treatment can be interpreted as the average increase in outcomes for 1-day increase in age at the start of treatment. Interaction between diet and gemcitabine was excluded from models due to nonsignificance. The two-way and three-way interactions between sex and treatments (diet, gemcitabine) are nonsignificant.

**FIGURE 2:**
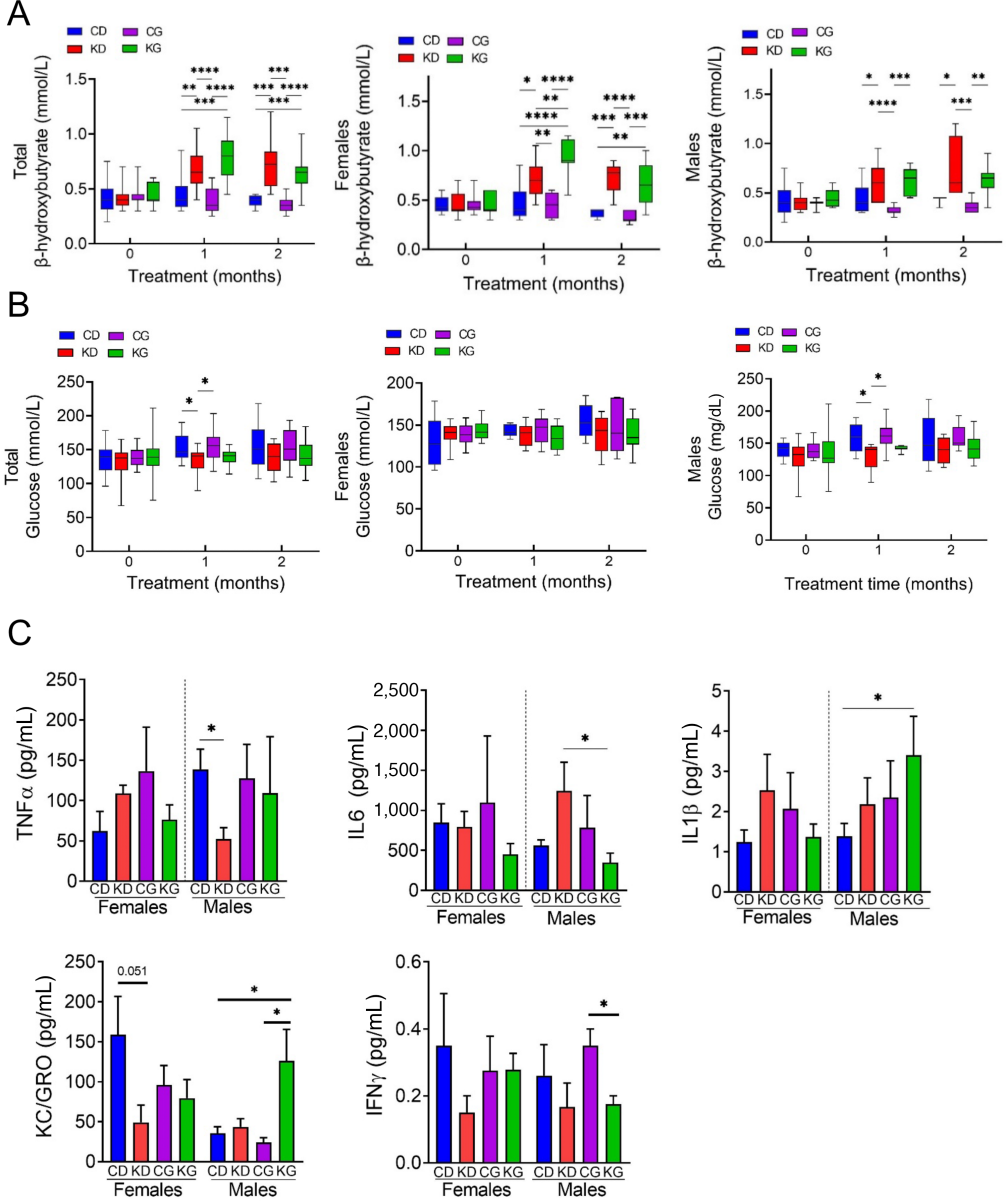
Metabolic changes of KPC mice fed KD with or without chemotherapy. **A,** Blood β-hydroxybutyrate levels at baseline and after each month in each group in total cohort (left), females only (center), and males only (right) are shown. *, *P < 0.05*. **B,** Circulating levels of nonfasting glucose in total cohort (left), females only (center), and males only (right) are shown. **C,** Cytokines TNFα, IL6, IL1β, KC/GRO, and IFNγ were measured in serum obtained from KPC mice fed a CD, KD, CG, or KG at euthanasia. Results are expressed as mean ± SD. *, *P < 0.05*.

Mice fed a KD, alone or in combination with gemcitabine, showed significantly increased blood ketones compared to mice fed a CD or CG (Fig. 2A). The β-hydroxybutyrate levels in the KD and KG groups remained elevated throughout the study. The increase in β-hydroxybutyrate levels were observed in both female and male mice fed a KD or KG (Fig. 2A). In contrast, glucose levels were significantly higher in the CD and CG groups when compared with KD only at 1 month (Fig. 2B). When disaggregated by sex, such effect was only observed in males (Fig. 2B).

Furthermore, because KDs have been shown to exert anti-inflammatory effects (29), we assessed the levels of several proinflammatory cytokines in the serum of male and female KPC mice at endpoint. In males, there was a decrease of TNFα in KD compared with CD and a decrease in IL6 in the KG group when compared with KD. In addition, higher levels of IL1β were observed in KG males compared with CD. In males, higher levels of KC/GRO were observed in the KG group compared with CD and CG groups. On the other hand, in females, no significant changes in serum cytokines were observed among the CD, KD, CG, and KG groups (Fig. 2C). Moreover, no significant differences, in both males and females, were observed in IFNγ_,_ IL10, or MCP-1 levels among the groups (Supplementary Fig. S4).

### Evaluation of Cellular Mechanisms of a KD Related to Tumor Growth

**FIGURE 3:**
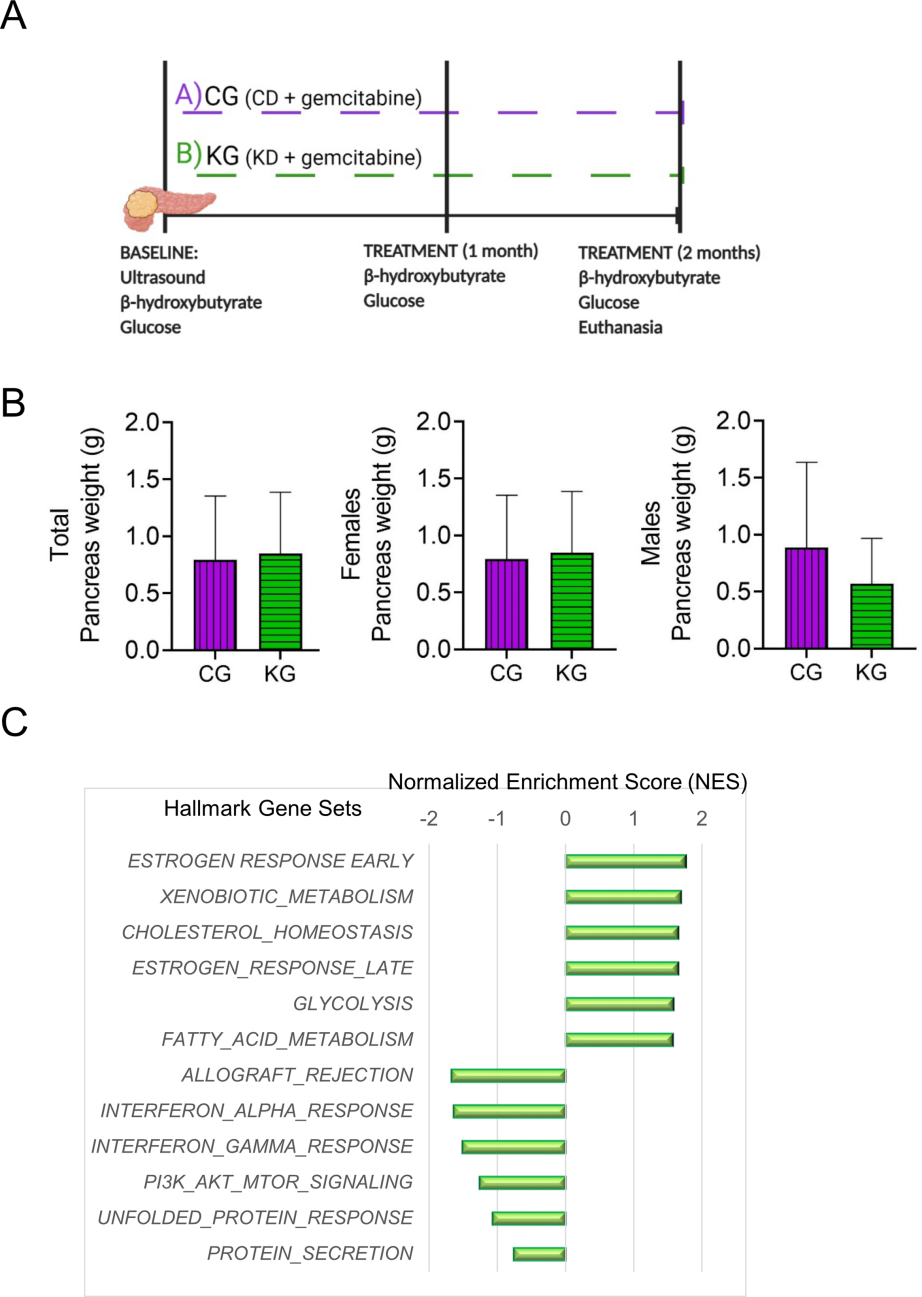
Hallmarks of pathways enriched following a KG treatment. **A,** Schematic outline of the mechanistic study design. **B,** Pancreatic tumor weight in CG and KG groups following 2 months of treatment. **C,** Top six hallmark gene sets identified as increased or decreased in pancreatic tumors isolated from KG-treated mice compared with pancreatic tumors isolated from CG-treated mice, using all differentially expressed genes (*P* < 0.05).

To elucidate the cellular mechanisms underlying the beneficial effects of a KD plus gemcitabine on pancreatic tumors, we conducted a study in which male and female KPC mice bearing pancreatic tumors (3 months old) were treated with either CG or KG for 2 months (Fig. 3A). We chose CD plus gemcitabine as our control group to specifically depict the contribution of a KD to the effect. After 2 months of treatment, no differences in the weights of the pancreas and/or tumors were observed between CG- and KG-treated mice (Fig. 3B).

To investigate whether KD plus gemcitabine induces changes in PDAC tumors in females that would suggest general antitumor activity, we initially performed an RNA-Seq analysis followed by HALLMARK gene set enrichment analysis (GSEA) on female pancreatic tumors obtained from KG or CG mice after 2 months of treatment. KG treatment was broadly associated with increased changes in the expression of genes involved in early and late estrogen response, xenobiotic metabolism, glycolysis, and fatty acid metabolism. In contrast, KG treatment was associated with decreased changes in the expression of genes involved in allograft rejection, IFN alpha and gamma response, PI3K-AKT-mTOR as well as unfolded protein response (Fig. 3C).

### A KD Inhibits the AKT and ERK Pathways in KPC Mice

**FIGURE 4:**
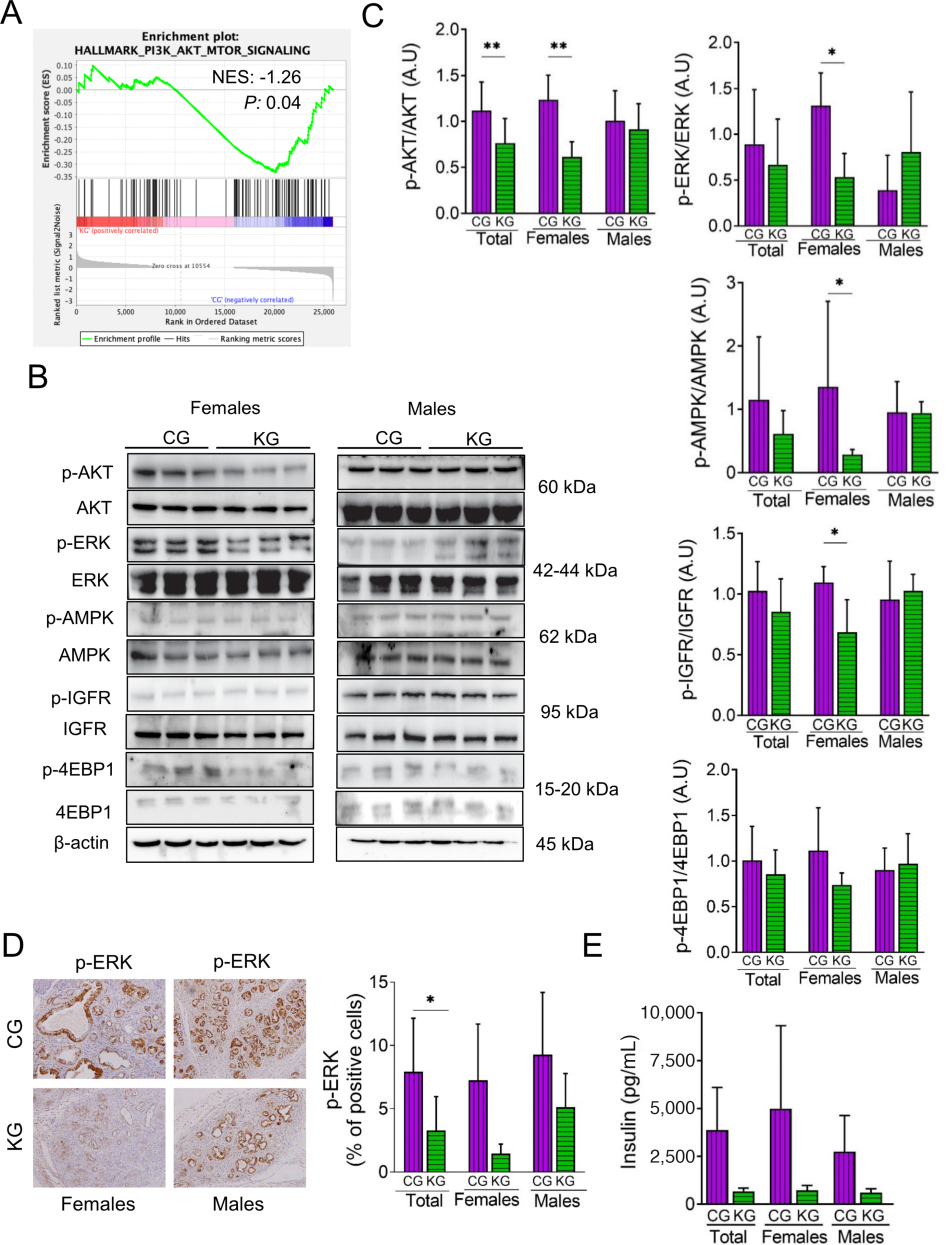
A KG reduces AKT and ERK activation in KPC mice. **A,** GSEA was conducted on RNA-seq data obtained from pancreatic tumors of KG- and CG-treated KPC mice. The enrichment plot for the PI3K_AKT_MTOR gene set downregulated by KG treatment (relative to CG) is depicted. Normalized enrichment score (NES) and nominal *P* value (*P*) were provided according to GSEA **B:** Immunoblots of p-AKT, AKT, p-ERK, ERK, p-AMPK, AMPK, p-IGFR, IGFR, p-4EBP1, and 4EBP1 in pancreatic tumor homogenates isolated from CG- and KG-treated KPC mice following 2 months of treatment. Loading control: β-actin. **C,** Bands were quantified and results are expressed as % control; *, *P < 0.05*; **, *P < 0.01*. **D,** IHC for p-ERK were performed on KPC tumor sections and photographs were taken at 20 × magnification Representative images are shown. Results were expressed as percent of p-ERK+ cells ± SD per × 20 field. *, *P < 0.05*. **E,** Insulin levels were measured in serum obtained from KPC mice fed a CG or KG for 2 months.

Two pathways commonly activated in PDAC are PI3K-AKT-MTOR and Kras/MAPKs (30, 31). GSEA data indicated that PI3K-AKT-MTOR was one of the pathways downregulated in the KG group, compared with CG (Fig. 4A). Thus, we validated these data by assessing the activation status of key proteins in the PI3K/Akt/mTOR, as well as the Raf/MEK/ERK pathways by immunoblot. Although there was no significant difference in AKT, ERK, or AMPK phosphorylation in pancreatic tumors between KG and the other groups in the survival study (Supplementary Fig. S5), KG treatment significantly reduced AKT, ERK, IGFR, and AMPK phosphorylation in pancreatic tumors of female, but not male, KPC mice, compared with CG-treated mice, at 2 months of treatment (Fig. 4B and C). In contrast, no significant changes were observed in the expression levels of phosphorylated 4EBP-1 between the two groups (Fig. 4B and C). To confirm these results, we assessed ERK activation by IHC of tumor sections prepared from CG- and KG-treated female and male KPC mice. KG reduced p-ERK levels by 79% in females, compared with CG-treated mice (Fig. 4D; *P* < 0.08 for females).

Furthermore, because AKT activation can be regulated by insulin, we assessed serum insulin levels. Compared with CG-treated mice, after 2 months KG treatment reduced insulin levels by 85.5% in female and 78.2% in male KPC mice (Fig. 4E)*.*

### A KD Alters Glucose Metabolism in Pancreatic Tumors

**FIGURE 5:**
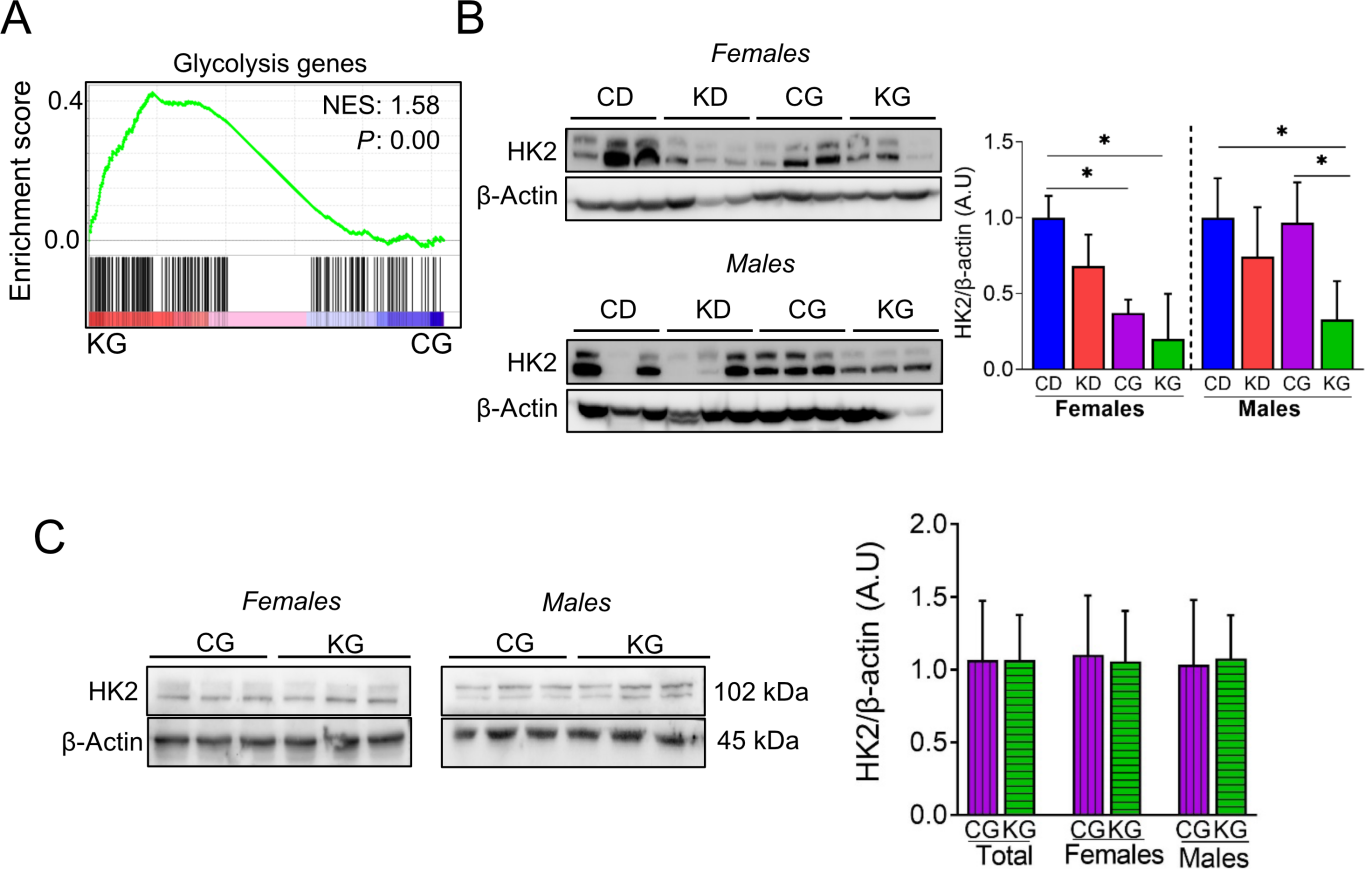
Effect of a KG on glycolytic pathway in pancreatic tumors. **A,** GSEA was conducted on RNA-seq data obtained from pancreatic tumors of KG- and CG-treated KPC mice. The enrichment plot for the Glycolysis gene set upregulated by KG treatment (relative to CG) is depicted. Normalized enrichment score (NES) and nominal *P* value (*P*) were provided according to GSEA. **B,** Immunoblots of HK2 in pancreatic tumor homogenates isolated from CD-, KD-, CG-, and KG-treated KPC mice at endpoint. Loading control: β-actin. Bands were quantified and results are expressed as % control; *, *P < 0.05*. **C,** Immunoblots of HK2 in pancreatic tumor homogenates isolated from CG- and KG-treated KPC mice following 2 months of treatment. Loading control: β-actin. Bands were quantified and results are expressed as % control.

Among many signatures, GSEA of differentially expressed genes in tumors from KG- and CG-treated female mice identified glycolysis signatures as highly affected (Fig. 5A). Thus, we assessed the expression levels of several enzymes linked to glucose metabolism in the pancreas/tumors of KPC mice. In both female and male KPC mice in the survival study, KG reduced hexokinase 2 (HK2) levels when compared with CD (Fig. 5B), but no changes were observed in animals euthanized at 2 months between KG and CG groups (Fig. 5C). Moreover, no significance differences were observed in the expression levels of LDH, PKM2, and PDH (Supplementary Fig. S6).

### A KD Affects the Concentrations of Fatty Acids in Pancreatic Tumors

**FIGURE 6:**
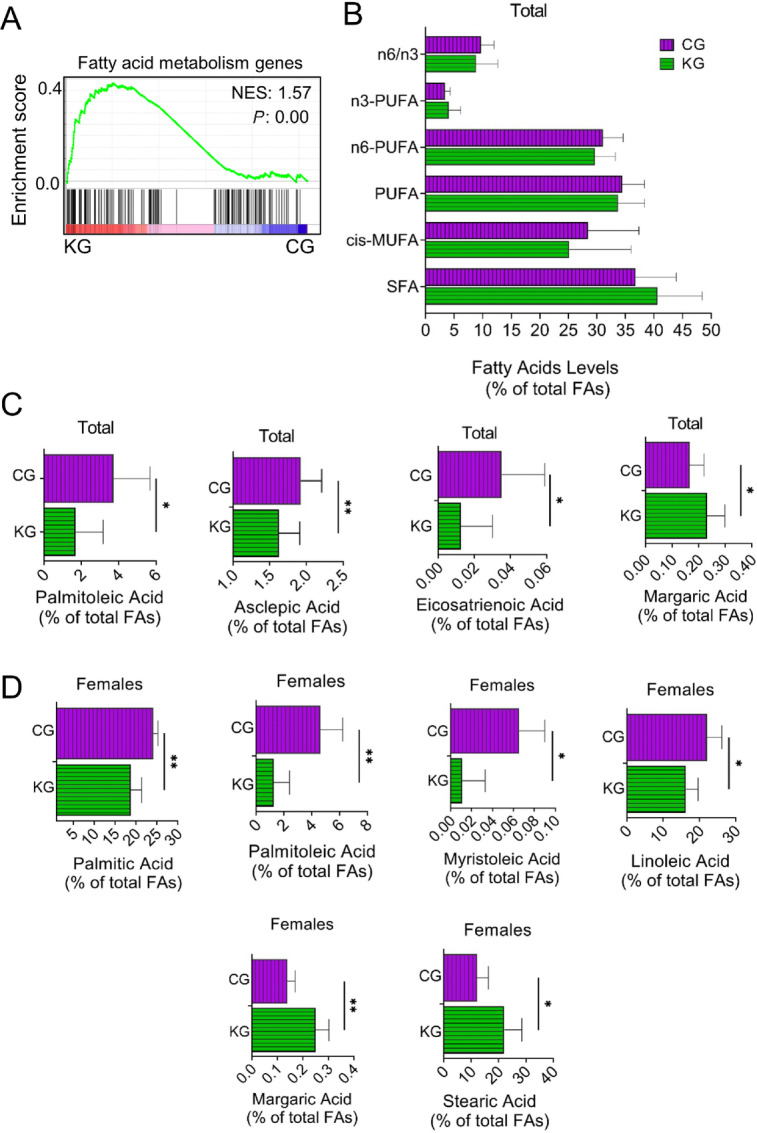
Effect of a KG on lipid metabolism in pancreatic tumors. **A,** GSEA was conducted on RNA-seq data obtained from pancreatic tumors of KG- and CG-treated KPC mice. The enrichment plot for the Lipid_Metabolism gene set upregulated by KG treatment (relative to CG) is depicted. Normalized enrichment score (NES) and nominal *P* value (*P*) were provided according to GSEA. **B,** Levels of SFAs, MUFA, PUFAs, as well as the n-6/n-3 fatty acid ratio in pancreatic tumors isolated from CG- and KG-treated KPC mice following 2 months of treatment. **C,** Concentrations (% of total fatty acids) of selected fatty acids (asclepic acid, palmitoleic acid, margaric acid, and eicosatrienoic acid) in pancreatic tumors homogenates isolated from CG- and KG-treated KPC mice (male and females combined) following 2 months of treatment. **D,** Concentrations (% of total fatty acids) of selected fatty acids (palmitic acid, margaric acid, myristoleic acid, palmitoleic acid, linoleic acid, and stearic acid) in pancreatic tumors homogenates isolated from CG- and KG-treated KPC female mice following 2 months of treatment. *, *P < 0.05*; **, *P < 0.01.*

GSEA of differentially expressed genes between KG and CG female KPC tumors identified fatty acid metabolism signatures as highly enriched in KG-treated mice compared with CG-treated mice (Fig. 6A). To understand more comprehensively, which fatty acids are affected in pancreatic tumors, we analyzed the fatty acid composition of tumors isolated from female and male KPC mice treated with a KG or a CG for 2 months. As shown in Fig. 6B, there were no significant differences in concentrations of total saturated fatty acids (SFA), total cis-monounsaturated fatty acids (*c*-MUFA), total n6-polyunsaturated fatty acids (n6-PUFA), and n3-PUFA in the pancreas of KG mice compared with CG mice. This holds true when separated by sex.

Interestingly, when examining changes of individual fatty acids between KG and CG, we observed that KG significantly reduced concentrations of asclepic acid (*cis*11–18:1), palmitoleic acid (*cis*9–16:1), and eicosatrienoic acid (20:3n-3), while increased margaric acid (17:0) content, compared with CG (Fig. 6C). Distinctively in female KPC mice, KG significantly reduced the concentrations of palmitic acid (16:0), myristoleic acid (*cis*9–14:1), palmitoleic acid (*cis*9–16:1), and linoleic acid (18:2n-6), and significantly increased the concentrations of margaric acid (17:0) and stearic acid (18:0) when compared with CG-treated females (Fig. 6D). No significant changes in any fatty acid concentrations were observed between KG- and CG-treated KPC male mice.

### A KD Plus Gemcitabine Alters Gut Bacterial Composition in KPC Mice

**FIGURE 7:**
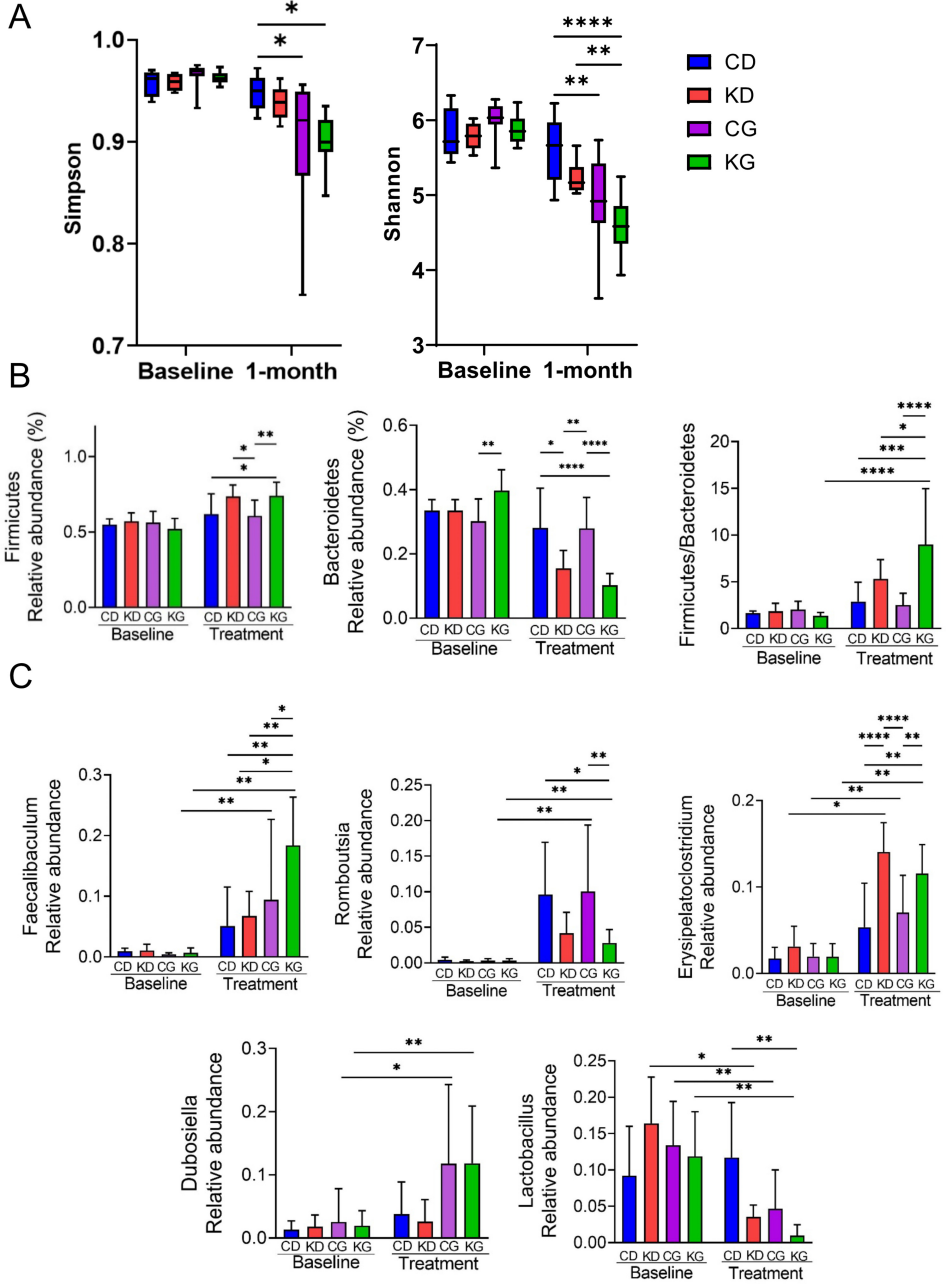
A KG affects the gut microbiota. **A,** Shannon and Simpson indexes were determined in CD, KD, CG or KG groups to evaluate the gut microbiota community diversity and richness among groups. *, *P < 0.05;* **, *P < 0.01.***B,** Levels of firmicutes, bacteroidetes and the ratio between firmicutes and bacteroidetes at baseline and after 1 month of treatment. *, *P < 0.05;* **, *P < 0.01.***C,** Levels of *Faecalibaculum, Romboutsia, Erysipelatoclostridium, and Dubosiella.*

Given that diet influences the composition of the gut microbiota, and the gut microbiota can affect PDAC growth and response to treatment (32, 33), we next performed 16S rRNA sequencing to evaluate the impact of a KD alone or in combination with GEM (KG) on the gut microbiota. For this purpose, we collected fecal samples at baseline (KPC mice fed chow diet, prior to dietary and/or chemotherapeutic treatments) and after 1 month of treatment with CD, KD, CG, or KG and assessed the α-diversity among groups. As expected, at baseline, there were no significant differences on the microbiota composition and/or diversity among the four groups. As shown by the Shannon and Simpson diversity indices, there were no significant differences on the microbiota diversity in the CD-fed group pre to post dietary intervention, but a significant difference was observed in both gemcitabine-treated groups (*P* < 0.001). When comparing mice fed a CD with those in the KG group, a significant difference was observed (*P* = 0.0003). Interestingly, a significant difference was observed when comparing animals fed a KD with those in the KG group, as depicted by the Shannon index (*P* = 0.0163; Fig. 7A).

We next analyzed the taxonomic components for all groups to confirm the specific changes of the microbial community. At the phylum level, Firmicutes and Bacteroidetes dominated the gut microbiota, and lower levels of Proteobacteria were detected. Compared with CD-fed mice, there was an increase in the relative abundance of Firmicutes in both KD-fed groups at 1 month of treatment. At 1 month of treatment, the ratio Firmicutes*/*Bacteroidetes was significantly higher for the KG (ratio = 9) group when compared with all others [CD (ratio = 2.9), KD (ratio = 5.3), CG (ratio = 2.5)] (Fig. 7B).

At the Genus level, all post-treatment groups increased the levels of *Faecalibaculum*, *Romboutsia*, and *Erysipelatoclostridium,* while reduced *Lactobacillus* levels*,* compared with baseline levels. *Romboutsia* levels were higher in the CD groups, while the increase in *Erysipelatoclostridium* was more apparent on the KD-fed animals. Interestingly, *Dubosiella* increased only in both GEM-treated groups. Of note, the levels of *Faecalibaculum* were significantly increased in the KG-treated mice when compared with all three other groups (Fig. 7C).

On the basis of the differences in microbial community composition among groups, we next performed a Bray–Curtis PCoA to define the similarity of species diversity among groups on OTU level (Supplementary Fig. S7A). Although there was a significant impact of treatment on microbial beta-diversity (PERMANOVA: F-value: 4.6619, *P* < 0.001), no significant changes due to sex were observed (PERMANOVA: F-value: 1.1273; *P* < 0.321; Supplementary Fig. S7B).

Finally, given that only KD plus gemcitabine increased overall survival, we aimed to identify some key species of bacteria that were differentially present in the KG group compared with KD or CG groups alone by performing a LEfSe analysis. The linear discriminant analysis (LDA) histogram was used to calculate the significant changes in the gut microbiota and interpret the degree of consistent difference of relative abundance between treatment groups. LDA results showed several discriminative features in the KG group (LDA>3.6, *P* < 0.05), compared with either KD or CG groups (Supplementary Fig. S8). The major species that were significantly increased in KG versus KD and KG versus CG include: genus*_Faecalibaculum,* class_*Erysipelotrichia* and order_*Erypiselotrichales* and family_*Eryspelotrichales. Moreover*, major species that were significantly decreased in KG versus KD and KG versus CG were order_*Lactobacillales*, family_*Lactobacillae, genus_Lactobacillus* phyllum*_Bacteroidetes,* order*_Bacteroidales,* class_*Bacilli* (Supplementary Fig. S8). Of note, increases in order_*Erypiselotrichales* and a decrease in order_*Lactobacillales, family_Lactobacillae*, genus_*Lactobacillus* was observed when comparing as the KD versus CD (Supplementary Fig. S8).

## Discussion

Dietary interventions hold promise in cancer treatment, including PDAC. Previous studies in animal models suggested that a KD is an effective adjuvant therapy for pancreatic cancer, yet the significance of the clinical benefit of KDs was limited because of the use of xenograft models, only one sex, or the use of small cohorts (12–14). We observed that in the clinically relevant KPC mouse model, mice fed a strict KD in combination with gemcitabine exhibited a significant increase in overall median survival, compared with KPC mice fed a CD, and this beneficial effect was superior in female mice compared with male mice. Although our linear regression model indicates that the effect of a KD plus gemcitabine is likely not sex dependent, benefiting both males and females, the survival curves suggest that the effect of a KD plus gemcitabine is somewhat more effective in females. Indeed, when disaggregating the data between females and males, the effect of a KD plus gemcitabine was significant in female KPC mice (60% increase in median overall survival), but not in male mice (28% increase in median overall survival). It is important to note that treatment with a KD alone had no effect on KPC survival, indicating that the dietary changes themselves were insufficient to cause the tumor responses.

Consistent with our findings, other investigators have recently evaluated the use of a KD in preclinical KPC allograft tumor models. For instance, Hopkins and colleagues observed that a KD rendered PI3K inhibitors, which are normally inactive against PDAC, effective in a KPC cell line–based orthotopic allograft tumors (14). In addition, Yang and colleagues recently showed that a KD synergized with a clinically relevant chemotherapeutic regimen of gemcitabine, nab-paclitaxel, and cisplatin, significantly increasing survival in subcutaneous KPC allograft tumors (17). Overall, these findings, together with our data, strongly indicate that a KD is an effective adjuvant dietary strategy for PDAC, and supports the initiated clinical trials (i.e.*,*NCT04631445), currently underway, to investigate its benefit in humans.

Mechanistically, the survival response to a KD plus gemcitabine appears to be multifactorial, including the inhibition of ERK and AKT pathways, regulation of fatty acid metabolism and the modulation of the microbiota. Interestingly, we noted some discrepancies between ours and Yang and colleagues's RNA-Seq data (17). For example, while allograft rejection, IFN alpha and gamma response gene sets were down regulated in our data, they noted the opposite. These discrepancies might be the result of the differences in tumor types used in the analysis (KPC tumors vs. allografts), differences of the tumor microenvironment, or the variances in the duration of KD intervention and other interventions (i.e., gemcitabine). Therefore, and as suggested by our RNA-Seq analysis, at this time, we cannot rule out that other mechanisms, including modulation of xenobiotic metabolizing enzymes or estrogen responses, could also contribute to the effect of a KD in PDAC.

Many features contribute to the reduced effectiveness of gemcitabine, including the dysregulation of signaling pathways related to cell metabolism (34), such as the insulin/IGF-1R, ERK, and PI3K/AKT pathways. For example, the PI3K/AKT pathway is aberrantly activated in multiple tumor types, regulating tumorigenesis, cancer metabolism, and drug resistance (35, 36). On the other hand, the deregulation of the ERK pathway is a signature of many epithelial cancers, including PDAC (37), whereas the upregulation of the insulin/IGF-1R pathway in PDAC occurs in over 70% of patients (38). Interestingly, compensatory upregulation of IGF-1R and ERK signaling limits the efficacy of select inhibitors, such as autophagy inhibitors, and their concurrent inhibition synergistically increases autophagy dependence and chloroquine sensitivity in PDAC (39). Therefore, the fact that a KD inhibits ERK, AKT, and IGFR activation might explain, at least in females, the survival benefit of its combination with gemcitabine.

Lipid metabolism is essential for cancer progression (40), with increased levels of specific fatty acids known to regulate pancreatic cancer progression (41). For example, Lien and colleagues recently showed that the upregulation of stearoyl-CoA desaturase, which synthesizes MUFAs from SFAs, is essential for cancer cells to grow (42). Interesting, they suggest that modifying the composition of the dietary fat could lead to higher tumor inhibitory effect. For instance, altering the KD fat composition, by using palm oil instead of lard as the source of fat, slowed tumor growth, by increasing tumor saturated fatty acid levels, lowering MUFAs and decreasing tumor stearoyl-CoA desaturase activity. Although we did not observe significant differences in overall SFAs or MUFAs between KG and CG groups, we observed a reduction in select MUFAs in the KG group compared with the CG group. Because the KD used in our study was mainly prepared with lard, it would be important to evaluate whether a KD from other fat sources that increase SFAs might provide an additional beneficial effect.

Several studies have also shown a positive association between higher consumption of certain fatty acids and pancreatic cancer risk. For example, high linoleic acid intake was shown to increase the risk of pancreatic cancer when compared with the individuals with the low linoleic acid intake (43). In a prospective nested case–control study, Yang and colleagues identified a fatty acid pattern using principal component analysis, associated with an increased risk of prostate cancer, which was characterized by higher levels of 14 and 16 carbon SFA and MUFA including myristic acid, palmitic acid, myristoleic acid and palmitoleic acid, along with low levels of α-linolenic acid (44). Interestingly, many of the fatty acids were reduced in pancreatic tumors following KG treatment, such as palmitic acid, myristoleic acid, palmitoleic acid, asclepic acid, and linoleic acid. Additional studies are warranted to validate whether one or more of these fatty acids could explain, in part, the beneficial effect of a KD in PDAC, and whether the modifying the type of fat used in the KD could lead a higher tumor inhibitor effect.

The gut microbiota is an emerging mediator of PDAC progression (45), with many strategies to modulate the gut microbiome in PDAC being actively explored (46). For example, the transplantation of human fecal microbes can affect PDAC tumor response by modulating the gut microbiota and the immune system (47). In addition, two bacterial communities (*Faecalibaculum and Lactobacillus)* have been recently documented to play a critical role regulating tumor growth. Zagato and colleagues identified that *Faecalibaculum rodentium*, belonging to the Erysipelotrichaceae family, was strongly underrepresented during the early phases of tumorigenesis in the *Apc*^Min/+^ mice compared with wild-type mice, and that it was responsible for inhibiting intestinal tumor cell proliferation (48). Moreover, *Faecalibaculum* can inhibit tumor growth in breast cancer models (49). On the other hand, bacteria belonging to the genus *Lactobacillus*, which are gut commensals with an ability to produce indoles from tryptophan (50), can drive suppression in the pancreatic tumor microenvironment promoting tumor growth (51). Our findings, showing that KG treatment leads to increased relative abundance of *Faecalibaculum* and the reduction of *Lactobacillus*, might provide a partial explanation of the beneficial effects of KD in combination with gemcitabine observed in KPC mice. Future investigations will determine whether the selective modulation of these bacteria can be used to improve the therapeutic response in PDAC.

In summary, a KD in combination with gemcitabine is beneficial as a treatment strategy for PDAC in KPC mice. The mechanisms by which KD plus gemcitabine increase survival response are multifactorial, including inhibition of ERK and AKT pathways, regulation of fatty acid metabolism and the modulation of the microbiota. These data in an autochthonous and clinically relevant mouse model strongly suggest that a KD should be evaluated concomitant to chemotherapeutic treatment in the clinical setting.

## Supplementary Material

Supplementary Table S1, Figures S1-S8 Supplementary Table S1. Experimental diets composition. Supplementary Figure S1. A ketogenic diet plus gemcitabine extends median overall survival in KPC mice. Supplementary Figure S2. Histopathological analysis of necrosis (C) in pancreatic tumor isolated from female and male KPC mice treated with CD, KD, CG or KD. Supplementary Figure S3. Histopathological analysis of fibrosis in pancreatic tumor isolated from female and male KPC mice treated with CD, KD, CG or KD. Supplementary Figure S4. IL-10, IFN-γ and MCP-1 levels were measured in serum obtained from female and male KPC mice fed a CD, KD, CG or KG at euthanasia. Supplementary Figure S5. Effect of a ketogenic diet plus gemcitabine on AKT and ERK activation at endpoint. Supplementary Figure S6. Effect of a ketogenic diet plus gemcitabine on glycolytic pathway in pancreatic tumors. Supplementary Figure S7. A ketogenic diet plus gemcitabine affects the gut microbiota. Supplementary Figure S8. A ketogenic diet affects the gut microbiota.
