# Correction: A Ketogenic Diet in Combination with Gemcitabine Increases Survival in Pancreatic Cancer KPC Mice

**DOI:** 10.1158/2767-9764.CRC-22-0510

**Published:** 2022-12-19

**Authors:** Natalia E. Cortez, Cecilia Rodriguez Lanzi, Brian V. Hong, Jihao Xu, Fangyi Wang, Shuai Chen, Jon J. Ramsey, Matthew G. Pontifex, Michael Müller, David Vauzour, Payam Vahmani, Chang-il Hwang, Karen Matsukuma, Gerardo G. Mackenzie

In the original version of this article (1), there is an error in the abstract. The macronutrient composition of both the ketogenic and control mouse diets was incorrectly reported in the abstract. The Materials and Methods section included the correct compositions and remains unchanged. The error has been corrected in the latest online HTML and PDF versions of the article. The authors regret this error.
