# Supplementary material for: A Ketogenic Diet in Combination with Gemcitabine Increases Survival in Pancreatic Cancer KPC Mice
Source: Cancer Res Commun. 2022 Sep 8;2(9):951–65. doi: 10.1158/2767-9764.CRC-22-0256 (PMC9648418; doi:10.1158/2767-9764.CRC-22-0256)
Supplement: Supplementary Table S1, Figures S1-S8 — Supplementary Table S1. Experimental diets composition. Supplementary Figure S1. A ketogenic diet plus gemcitabine extends median overall survival in KPC mice. Supplementary Figure S2. Histopathological analysis of necrosis (C) in pancreatic tumor isolated from female and male KPC mice treated with CD, KD, CG or KD. Supplementary Figure S3. Histopathological analysis of fibrosis in pancreatic tumor isolated from female and male KPC mice treated with CD, KD, CG or KD. Supplementary Figure S4. IL-10, IFN-γ and MCP-1 levels were measured in serum obtained from female and male KPC mice fed a CD, KD, CG or KG at euthanasia. Supplementary Figure S5. Effect of a ketogenic diet plus gemcitabine on AKT and ERK activation at endpoint. Supplementary Figure S6. Effect of a ketogenic diet plus gemcitabine on glycolytic pathway in pancreatic tumors. Supplementary Figure S7. A ketogenic diet plus gemcitabine affects the gut microbiota. Supplementary Figure S8. A ketogenic diet affects the gut microbiota. [file crc-22-0256-s01.pdf]

Supplemental Table 1. Experimental diets composition

Ingredient (g/kg diet)	Control	Ketogenic
Carbohydrates, of which	685	27.5
Sucrose	100	---
Maltodextrin	130	---
Corn starch	400	7.5
Vitamin Mix	20	20
Mineral Mix	35	---
Protein, of which	162.6	270.6
Casein	162	268
DL-methionine	2.6	2.6
Fat, of which	87.97	571.6
Lard	---	480
Soybean oil	87	90
**Fat in casein*	0.97	1.6
Cellulose	64	---
Tert-butylhydroquinone	0.014	0.130
Mineral Mix	---	20
Calcium phosphate dibasic	---	19
Calcium carbonate	---	8

A

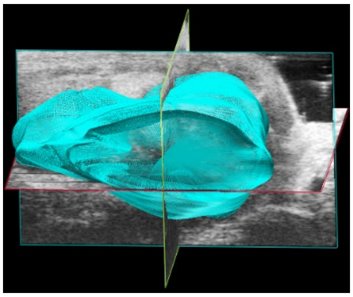

B

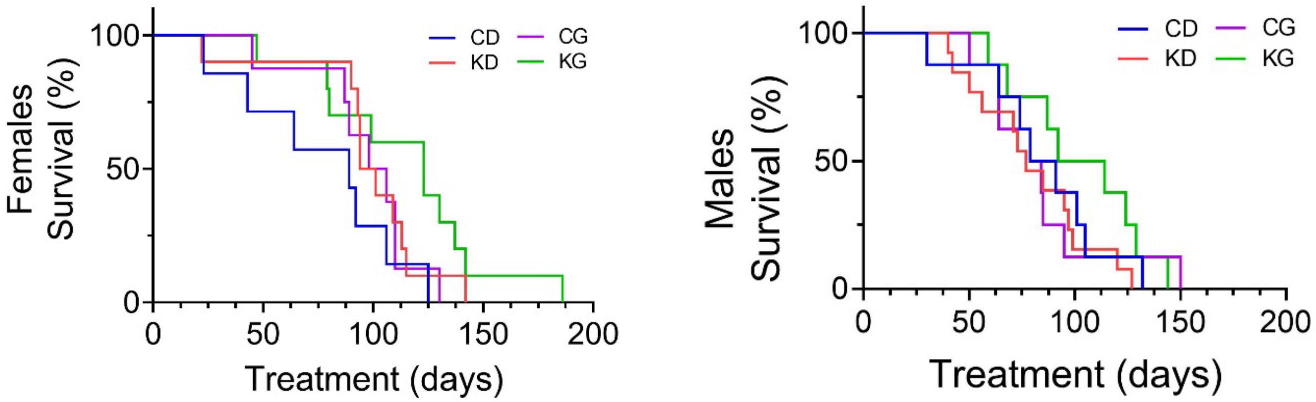

Figure S1. A ketogenic diet plus gemcitabine extends median overall survival in KPC mice. **A**: Representative ultrasound image of a pancreatic tumor in a KPC mouse at enrollment. **B:** Kaplan-Meier survival curves of male and female KPC mice fed a control diet (CD), ketogenic diet (KD), control diet plus gemcitabine (CG) or ketogenic diet plus gemcitabine (KG).

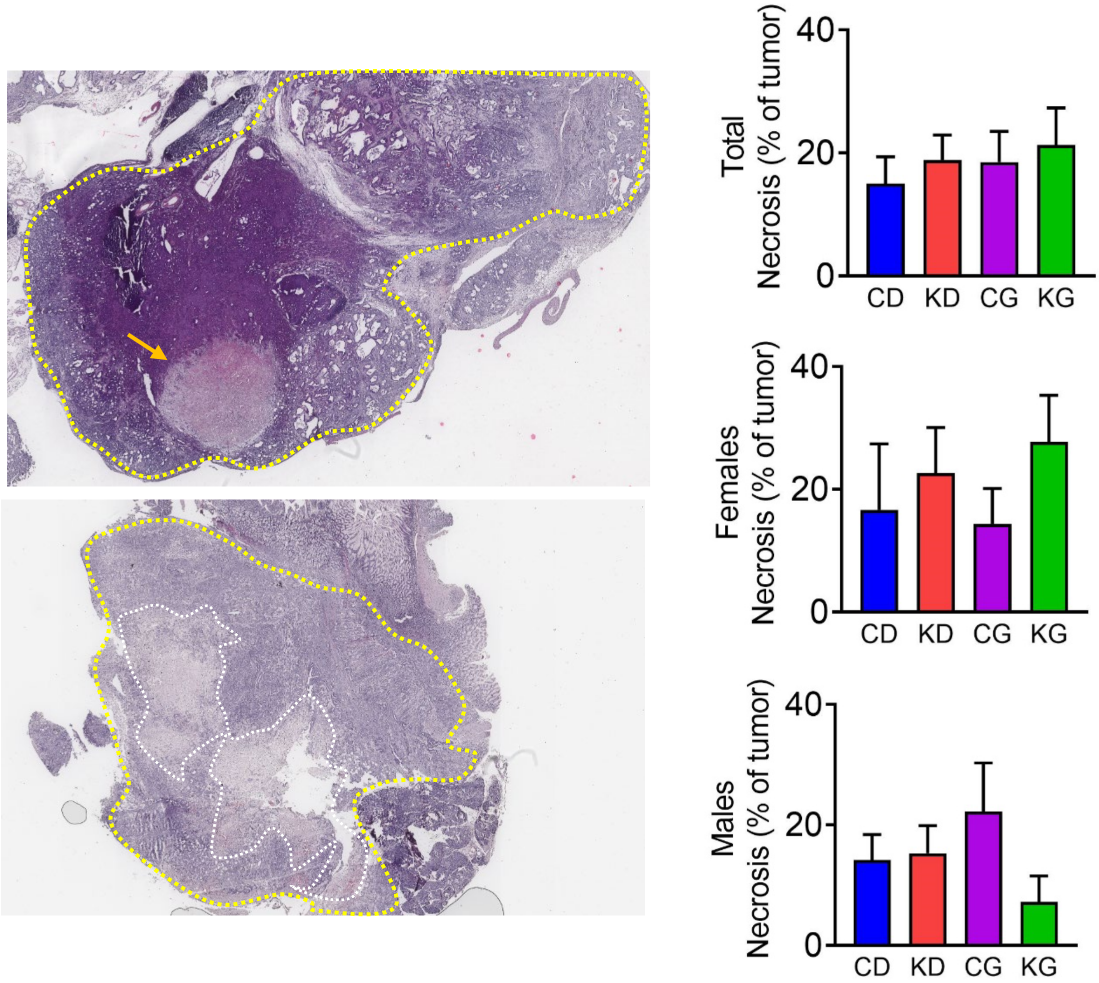

Figure S2: Histopathological analysis of necrosis (**C**) in pancreatic tumor isolated from female and male KPC mice treated with CD, KD, CG or KD. In the ketogenic diet treated groups, more tumor necrosis was noted overall. Representative images: (top) Control diet, (Bottom) Ketogenic diet. Yellow dotted line delineates total tumor surface area. Orange arrow highlights a discrete focus of tumor necrosis. White dotted line delineates more irregular and more extensive tumor necrosis. Hematoxylin and eosin-stained sections. All images digitally scanned at 20X original magnification.

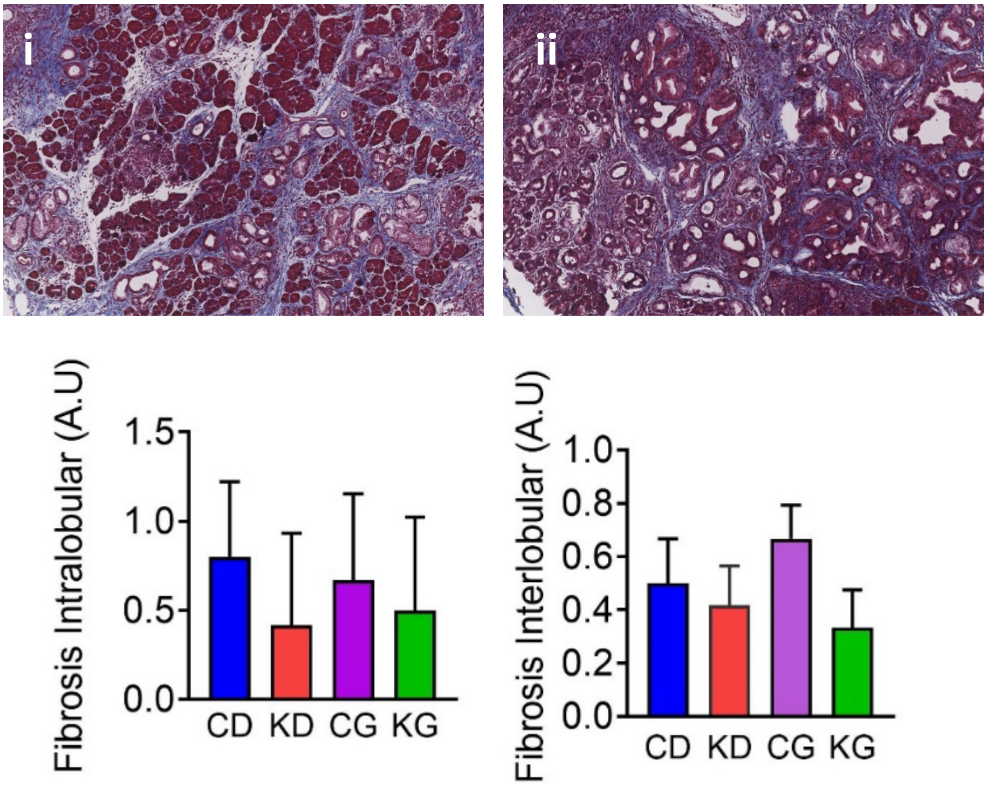

Figure S3: Histopathological analysis of fibrosis in pancreatic tumor isolated from female and male KPC mice treated with CD, KD, CG or KD. In all treatment groups, both intra- and interlobular fibrosis was observed in the background non-neoplastic pancreas. Representative images of Masson trichrome-stained sections: (i) Control diet, (ii) Ketogenic diet.. All images digitally scanned at 20X original magnification.

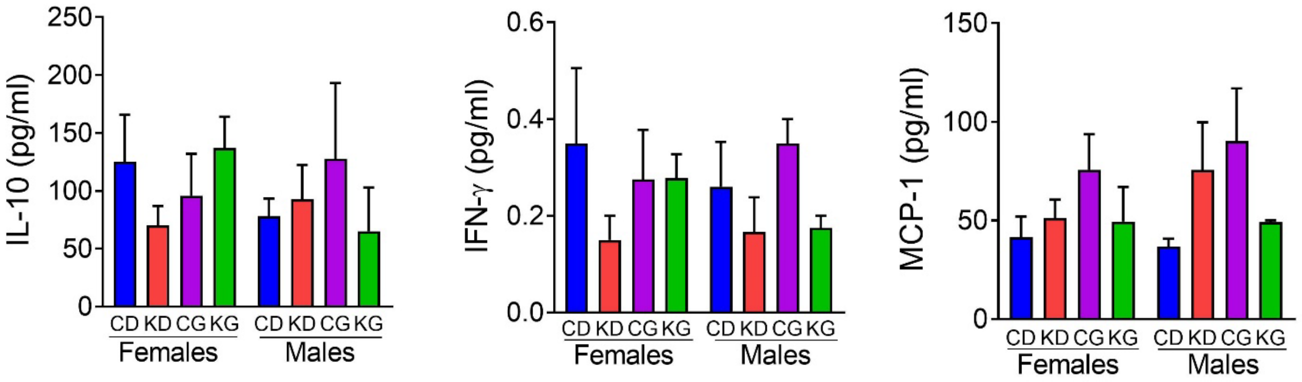

Figure S4: IL-10, IFN-γ and MCP-1 levels were measured in serum obtained from female and male KPC mice fed a CD, KD, CG or KG at euthanasia. Results are expressed as mean ± SD.

Supplemental Figure 5
Cortez et al

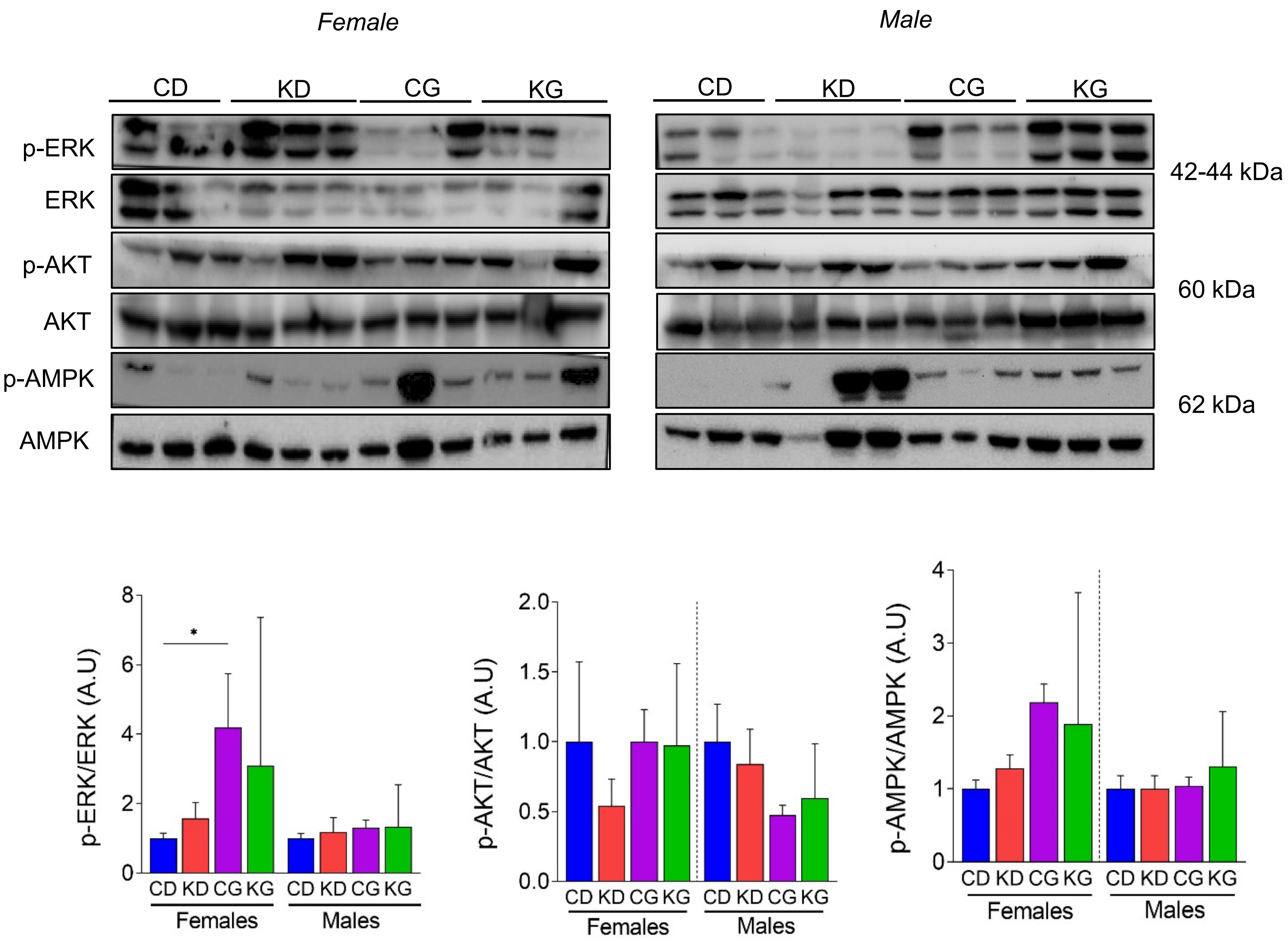

Figure S5. Effect of a ketogenic diet plus gemcitabine on AKT and ERK activation at endpoint. Immunoblots for p-AKT, AKT, p-ERK, ERK, p-AMPK and AMPK, in pancreatic tumor homogenates isolated from CD-, KD-, CG- and KG-treated female and male KPC mice when endpoint was reached. Bands were quantified and results are expressed as % control; **p<0.05.*

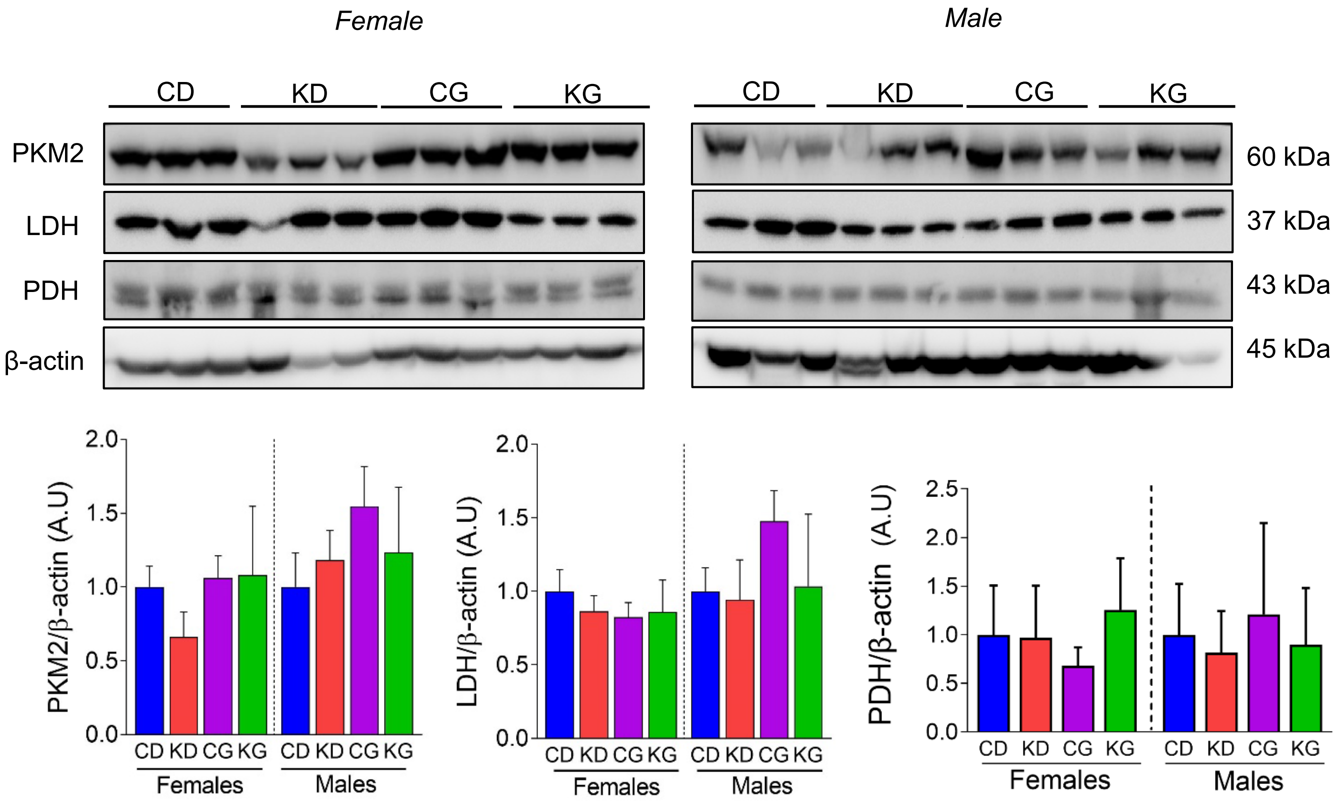

Figure S6. Effect of a ketogenic diet plus gemcitabine on glycolytic pathway in pancreatic tumors. Immunoblots for pyruvate kinase M2 (PKM2), Lactate dehydrogenase (LDH) and pyruvate dehydrogenase (PDH) in pancreatic tumor homogenates isolated from CD-, KD-, CG- and KG-treated female and male KPC mice at endpoint. Loading control: β-actin. Bands were quantified and results are expressed as % control; *$p<0.05$.

A

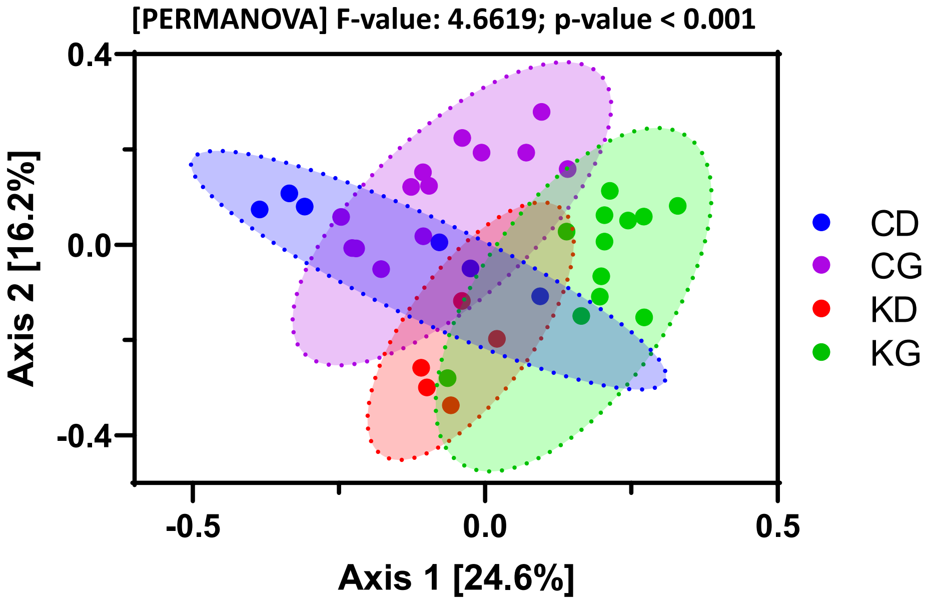

B

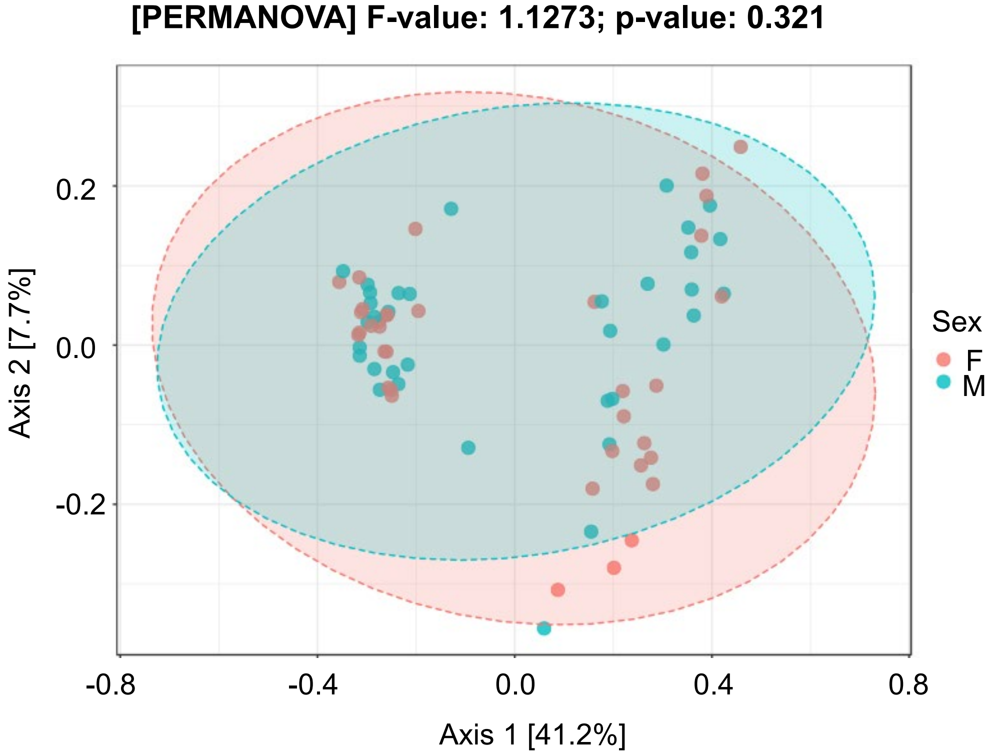

Figure S7: A ketogenic diet plus gemcitabine affects the gut microbiota. A-B: Principal component analysis (PCA). PC1 (axis 1) and PC2 (axis 2) represent the two most principal factors characterizing the bacterial profile among the 4 groups (**A**) or by sex (**B**) and their contribution rates (%) are shown on the axes.

Supplemental Figure 8
Cortez et al

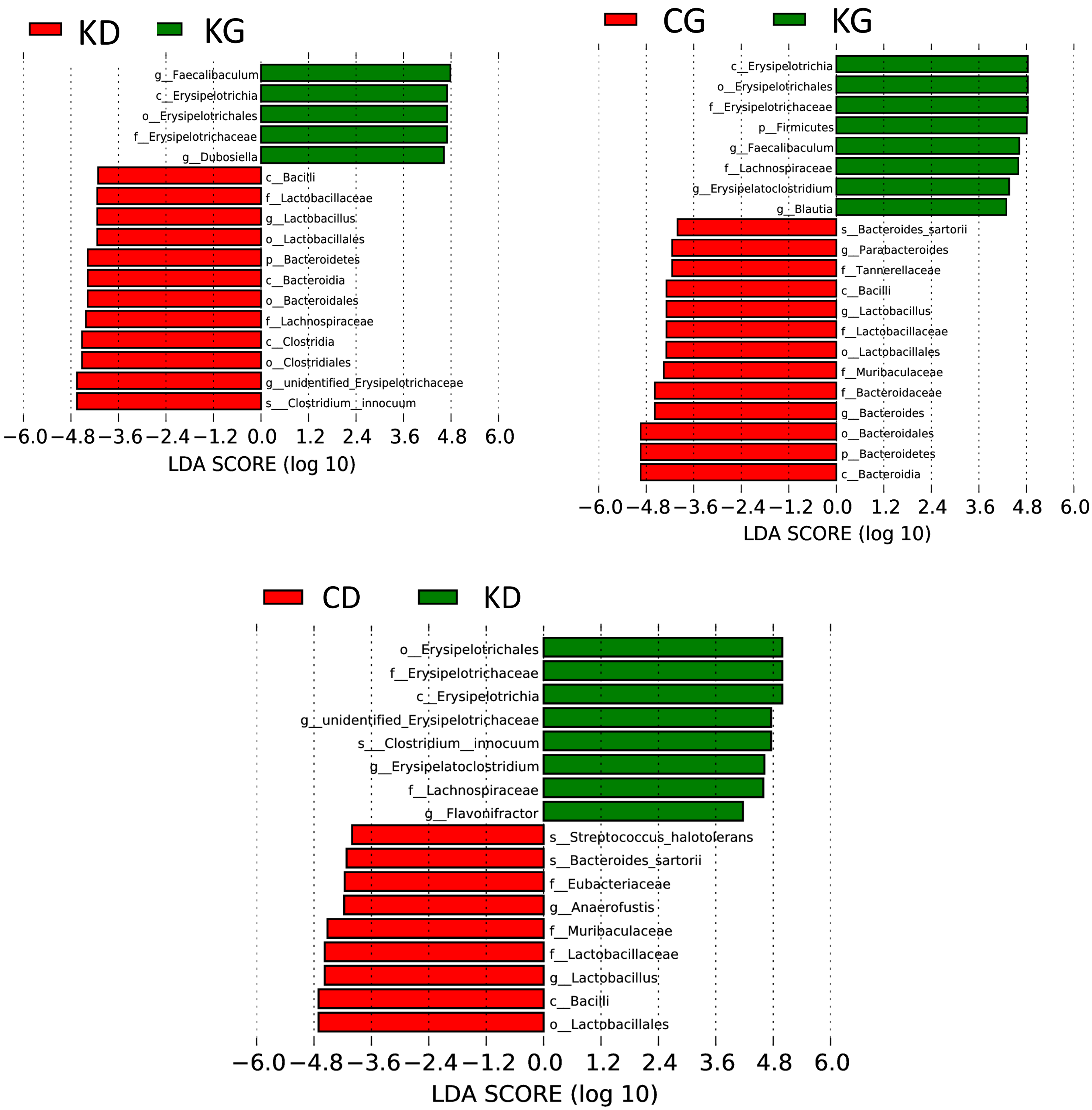

Figure S8: A ketogenic diet affects the gut microbiota. Distribution histogram of the LDA score determined by effect size (LEfSe). Bacterial taxa specifically enriched in groups with an LDA score > 3.6 are shown in the histograms. Comparisons after 1 month of diet/treatment were made between KG and KD (*upper left*), between KG to CG (*upper right*), and between KD and CD (*lower*) to highlight the bacteria taxa differentially enriched in KG-treated mice and in KD-treated mice.
